# Abscopal effect in maxillary sinus cancer: Insights from two case reports and a literature review

**DOI:** 10.1002/cnr2.1994

**Published:** 2024-02-13

**Authors:** Akihiro Sakai, Koji Ebisumoto, Hiroaki Iijima, Mayu Yamauchi, Daisuke Maki, Tsuyoshi Fukuzawa, Kenji Okami

**Affiliations:** ^1^ Department of Otolaryngology, Head and Neck Surgery Tokai University, School of Medicine Isehara Japan; ^2^ Department of Radiation Oncology Tokai University, School of Medicine Isehara Japan

**Keywords:** abscopal effect, head and neck cancer, immune checkpoint inhibitors, maxillary sinus cancer

## Abstract

**Background:**

The abscopal effect is a rare phenomenon in which localized radiation therapy triggers tumor reduction in nontargeted areas. Although this phenomenon has been observed in various cancer types, it remains infrequent and not fully understood.

**Case:**

Two patients with maxillary sinus cancer with distant metastases were treated with radiotherapy after immune checkpoint inhibitor (ICI) therapy. The patients demonstrated abscopal effects following ICI therapy and radiotherapy, showing shrinkage in metastatic areas not directly targeted by radiation.

**Conclusion:**

This report was reviewed to examine the synergistic effects of ICI and radiotherapy and to identify optimal strategies to enhance the abscopal effect in clinical practice. It has also touched on various ongoing debates and clinical trials aimed at understanding and exploiting this effect to improve cancer treatment. The exact mechanisms and optimal treatment protocols remain areas for future research.

## 1 INTRODUCTION

The abscopal effect is a rare phenomenon observed in the treatment of metastatic cancer, in which local irradiation causes the tumor to shrink or disappear in areas not directly targeted by treatment.^1^ The abscopal effect is thought to be caused by immune system activation. Several mechanisms have been proposed, including T cell activation,^2^ the release of cytokines,^3^, ^4^ immune responses to tumor‐associated antigens (TAA),^5^, ^6^ immunogenic cell death (ICD),^7^, ^8^, ^9^ and exosomes.^6^ Although several theories have been proposed, the exact mechanism underlying the abscopal effect is not fully understood.

The abscopal effect has been observed in various types of cancer, including melanoma, breast cancer, and lung cancer; however, it is considered infrequent.^10^ However, since the advent of immune checkpoint inhibitors (ICIs), reports on this phenomenon have increased.^11^ Here, we report two cases of maxillary sinus cancer treated with ICI followed by radiotherapy, in which we experienced what we considered an abscopal effect, including a literature review.

## 2 CASE REPORTS

### 2.1 Case 1

**FIGURE 1:**
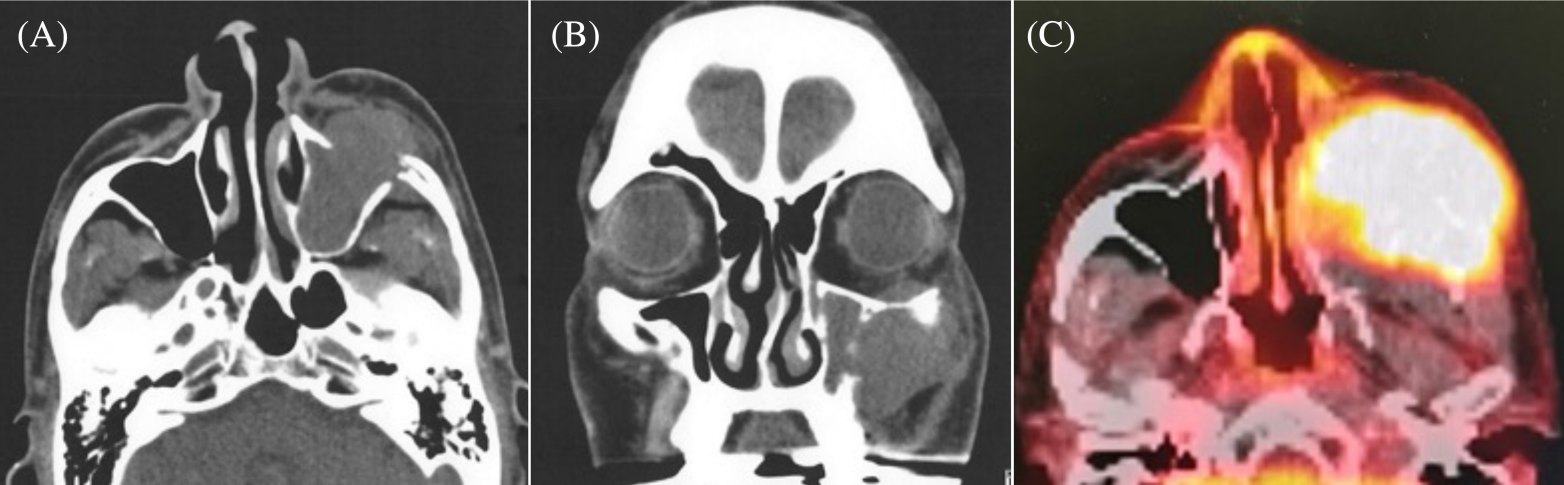
Computed tomography (CT) at initial presentation. (A) Axial view showing a tumor occupying the maxillary sinus with bony destruction of the anterior wall. (B) Coronal image showing intraocular extension with bone destruction in the superior and lateral walls. (C) Positron emission tomography/CT showing intense uptake in the same lesion.

**FIGURE 2:**
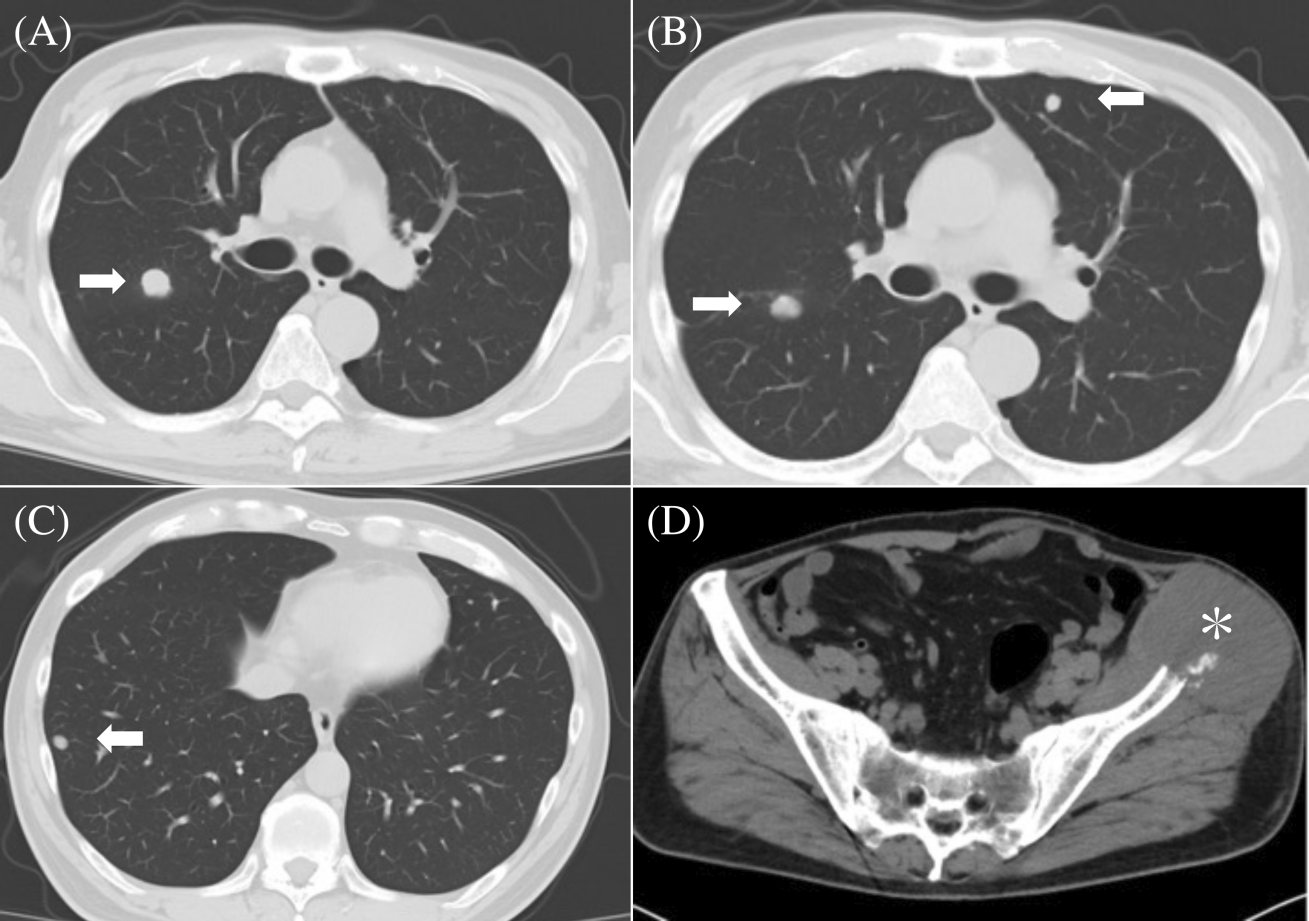
Computed tomography imaging after five courses of nivolumab administration. (A–C) Progression of multiple lung metastases (white arrows) and (D) left iliac bone metastases (asterisks) are observed.

**FIGURE 3:**
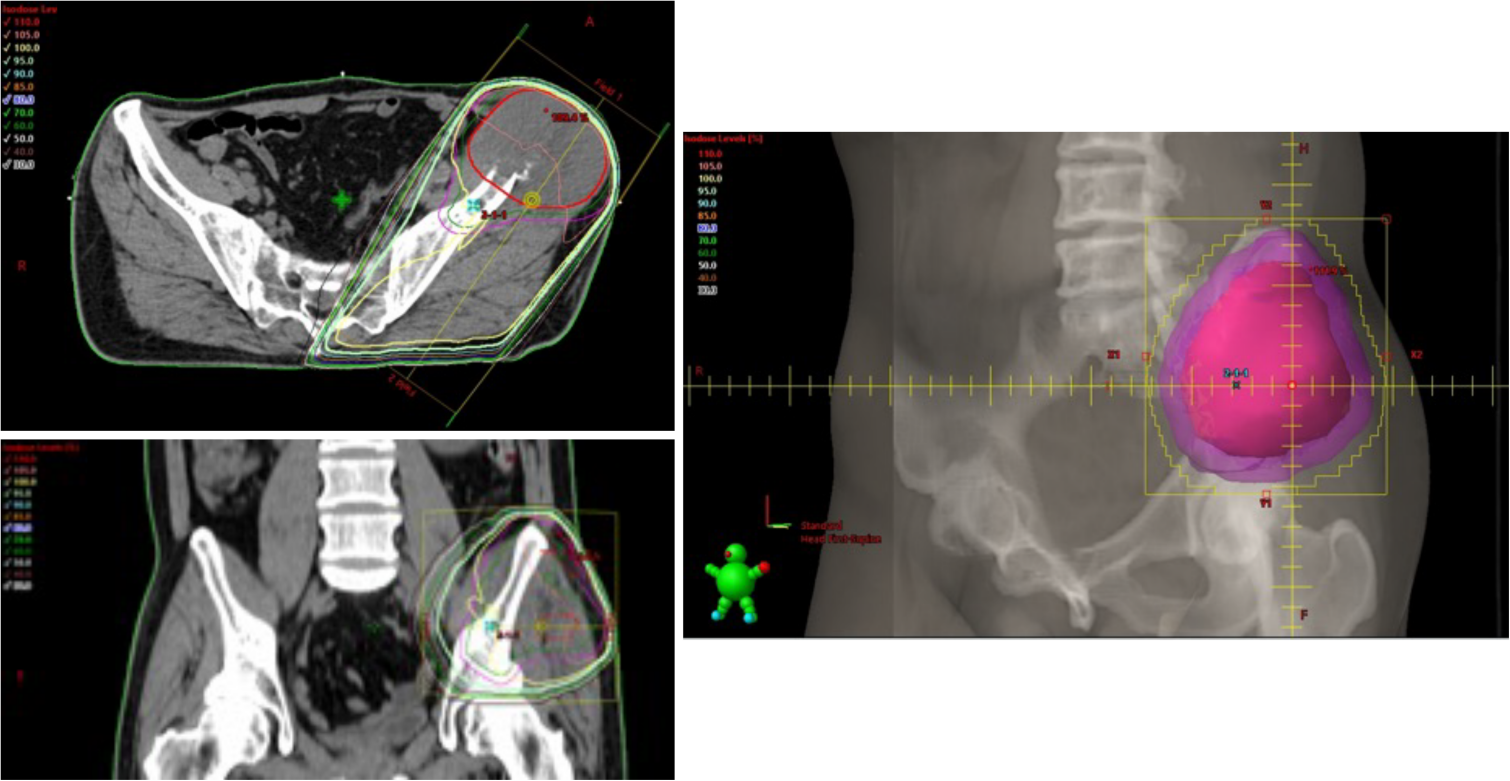
Radiation treatment plan. Palliative radiotherapy to pelvic bone metastases of a total dose of 20 Gy (4 Gy × 5 fractions) is administered. In the application of three‐dimensional conformal radiotherapy (3DCRT), the Gross Tumor Volume (GTV) is defined as the pelvic bone metastases, with the Clinical Target Volume (CTV) being GTV plus 5 mm, and the Planning Target Volume (PTV) set as CTV plus an additional 5 mm.

**FIGURE 4:**
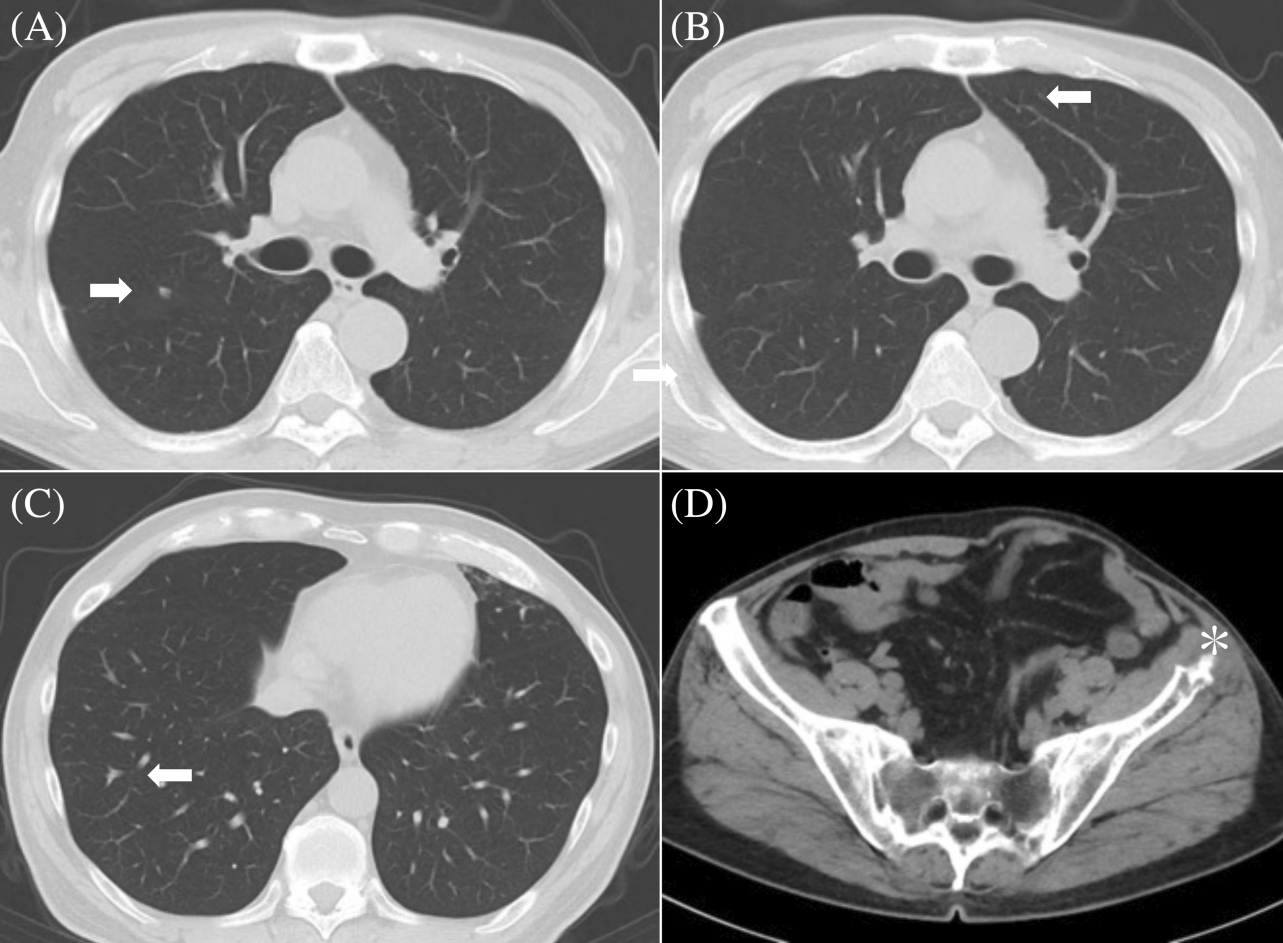
Computed tomography imaging 1 month after radiotherapy. (A, B, C) Multiple lung metastases (white arrows) and (D) iliac bone metastases (asterisks), shown in Figure 2, are observed with remarkable shrinkage.

A 51‐year‐old male patient presented Tokai University Hospital in January 2022 with complaints of left buccal pain. Imaging studies (computed tomography (CT), positron emission tomography (PET)/CT scan) and biopsy were performed, and he was diagnosed with maxillary sinus cancer (squamous cell carcinoma [SCC], T4N0M0 [Figure 1]). He underwent a left total maxillectomy, left neck dissection, and maxillary reconstruction with a left rectus abdominis muscle flap in March 2022. After surgery, the patient received postoperative concurrent chemoradiotherapy (66 Gy (2Gy × 33 fractions) and cisplatin 80 mg/m^2^). However, 3 months after treatment, CT scan revealed multiple nodular lesions in the lungs, which were diagnosed as lung metastases. PD‐L1 testing of the primary lesion was performed and the Combined Positive Score (CPS) was <1. Therefore, the patient received five courses of nivolumab, an ICI. However, lung metastasis worsened and additional metastasis to the pelvis developed (Figure 2). The patient experienced severe pain; therefore, palliative radiotherapy to pelvic bone metastases with a total dose of 20 Gy (4 Gy × 5 fractions) was administered in November 2022 (Figure 3). Rapid symptomatic improvement was observed. CT scan 1 month after radiotherapy showed significant shrinkage of both lung and pelvic metastases (Figure 4). The patient has continued nivolumab therapy for about a year now, maintaining a partial response (PR) without any signs of regrowth.

### 2.2 Case 2

**FIGURE 5:**
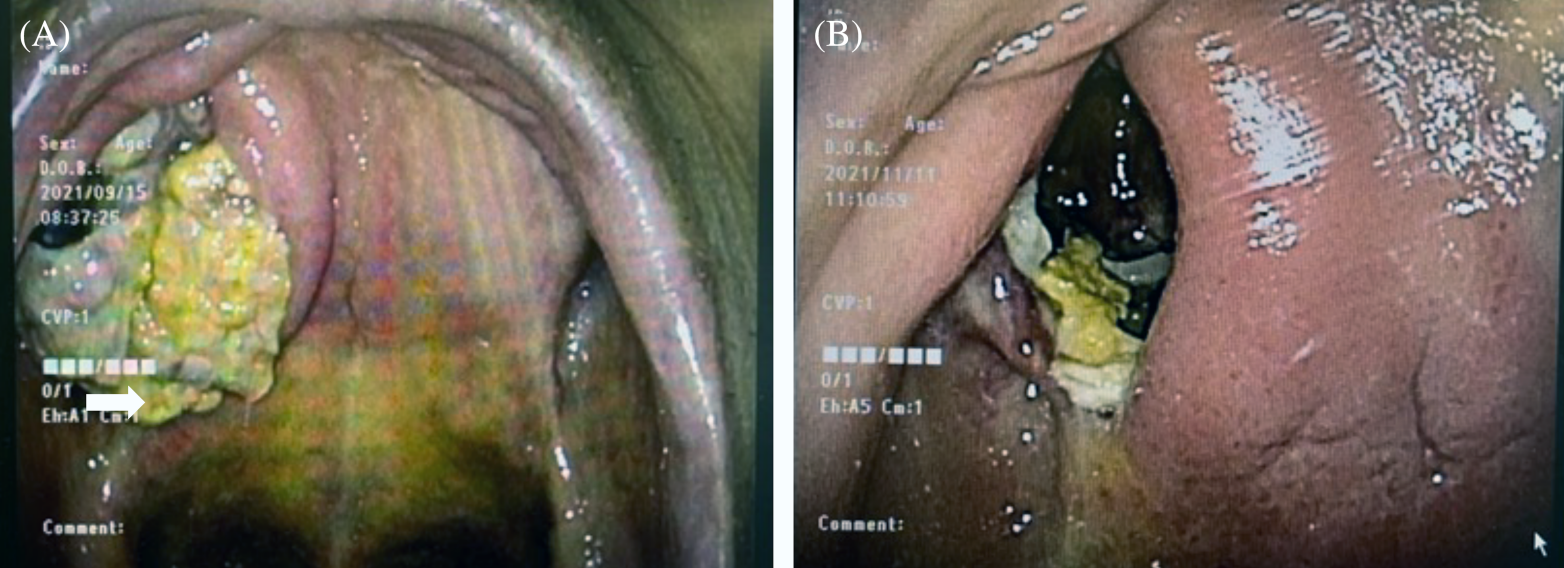
Oral findings (A) before and (B) after S‐1 administration. (A) Prior to treatment, the tumor in the maxilla had extended into the palate with bone destruction. (B) The tumor is significantly shrunk by S‐1 administration.

**FIGURE 6:**
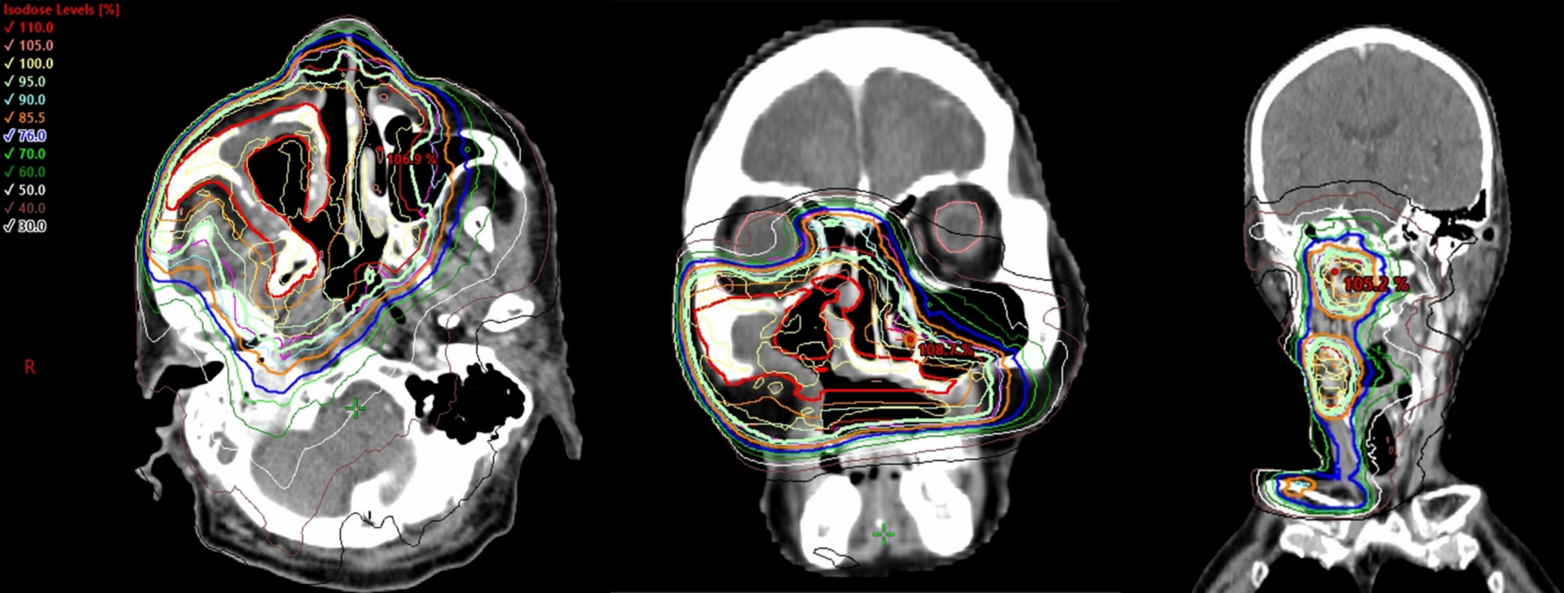
Radiation treatment plan. Radical radiotherapy (66 Gy: 2Gy × 33 fractions) is performed on the maxilla and neck to improve the local quality of life. We define the Gross Tumor Volume (GTV) as the primary tumor and metastatic lymph nodes, with high, intermediate, and low‐risk Clinical Target Volumes (CTVs) sequentially expanded by 5 mm from GTV, and their corresponding Planning Target Volumes (PTVs) receiving 66, 59.4, and 52.8 Gy respectively using Volumetric Modulated Arc Therapy (VMAT) with Simultaneous Integrated Boost (SIB).

**FIGURE 7:**
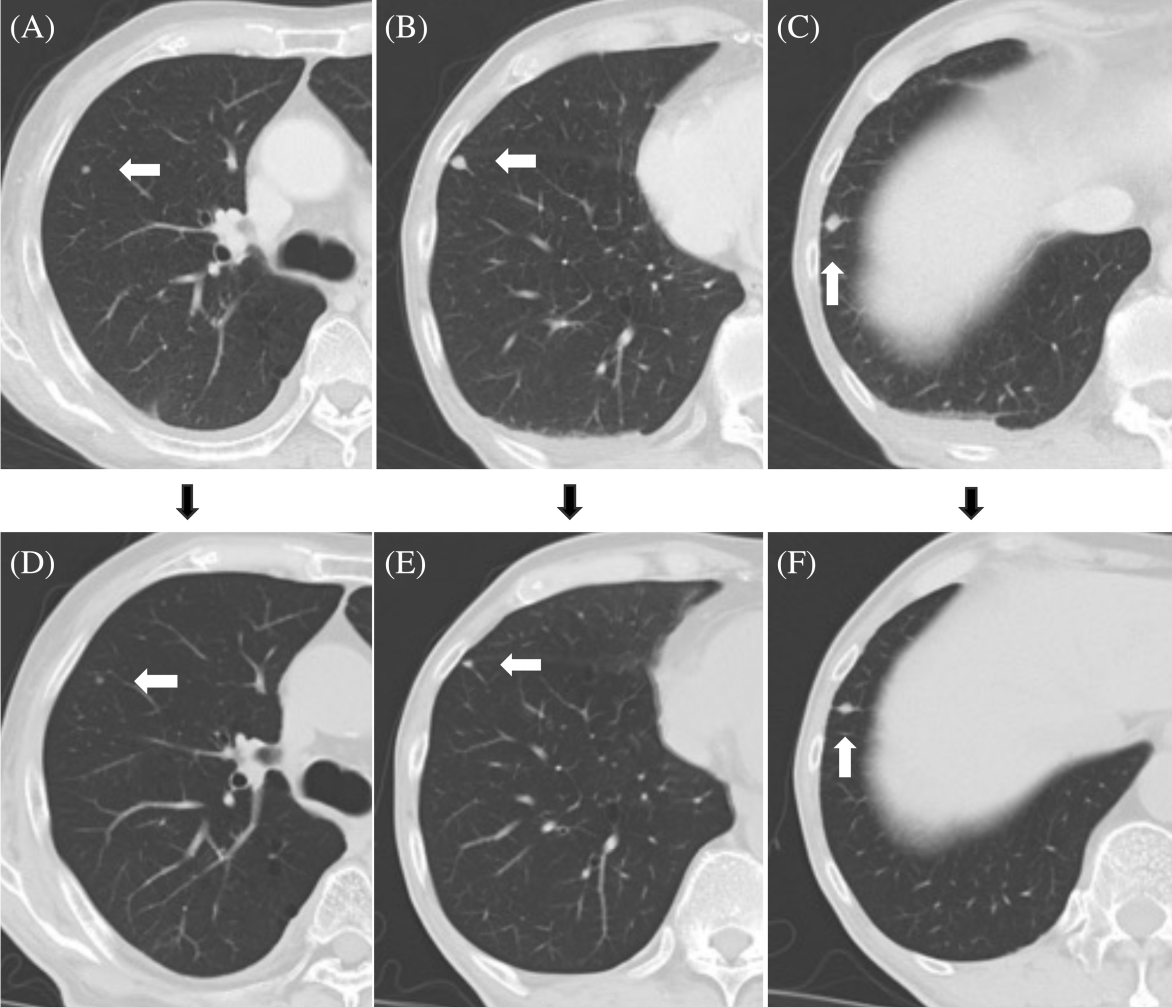
Computed tomography imaging (A–C) before and (D–F) after radiation. Lung metastases observed before radiotherapy are slightly reduced by radiotherapy of the primary tumor.

A 72‐year‐old man was referred to Tokai University Hospital in November 2017 with the chief complaint of right nasal obstruction, right facial numbness, and right cervical lymphadenopathy. Imaging studies and biopsy were performed, and he was diagnosed with maxillary sinus cancer and lung metastasis (SCC, T4aN1M1). Patients received six cycles of initial chemotherapy, comprising cisplatin (70 mg/m^2^), 5‐fluorouracil (700 mg/m^2^), and cetuximab (400–250 mg/m^2^), followed by a secondary chemotherapy regimen with paclitaxel (80 mg/m^2^) and cetuximab (250 mg/m^2^). The primary and neck lesion showed a PR, while the lung metastasis achieved a complete response (CR). This treatment effect was maintained for 18 months. However, progressive disease (PD) was observed at the primary site in August 2019, prompting a switch to nivolumab therapy. PD‐L1 testing was conducted, but it was impossible to measure due to the inadequacy of the sample. The patient underwent four courses of nivolumab; however, the disease continued to progress. A rechallenge with paclitaxel and cetuximab resulted in tumor shrinkage, and a maintained PR for about 1 year. Unfortunately, in September 2021, the patient suffered from severe bleeding and pain due to an advanced tumor in the maxillary region. CT scan revealed that the primary site was PD, the neck lesion was SD, and recurrence of multiple lung metastases was noted. Therefore, the treatment was switched to tegafur, 5‐chloro‐2,4‐dihydro‐pyrimidine, and potassium oxonate (S‐1100 mg). Consequently, the size of the primary lesion in the maxilla significantly reduced (Figure 5). In contrast, lung metastasis did not shrink. Tumor shrinkage with S‐1 enabled local control with radiotherapy. Given that the lung metastasis was nonaggressive, the patient was recommended and approved for local radiotherapy. This decision was based on the consideration that any potential local regrowth could significantly diminish the patient's quality of life. Therefore, radiotherapy (66 Gy (2Gy × 33 fractions)) was performed on the maxilla and neck to improve the local quality of life (Figure 6). After treatment, the local disease showed a complete response. Simultaneously, shrinkage of lung metastases in non‐irradiated areas was observed (Figure 7). The patient has been under observation without treatment for 20 months. During this period, the primary and neck lesions have shown a CR, while the lung metastasis has maintained a PR.

## 3 DISCUSSION

### 3.1 Synergy with immune checkpoint inhibitors

Abscopal effects are rare; however, since the advent of ICIs, the number of reports on abscopal effects has increased, especially in cases combining radiotherapy and ICIs.^12^ Liu et al. noted that only 46 clinical cases of abscopal effects caused by radiotherapy alone were reported between 1969 and 2014.^8^ They suggested that the overall low incidence of the abscopal effect was due to the inability of radiotherapy alone to overcome the immune tolerance mechanisms of the tumor cells. However, Oshiro et al. found that, in 2021 alone, there were 21 case reports of the abscopal effect after radiotherapy.^11^ This increasing trend in the abscopal effect may be due to the synergistic effect of the combination of radiotherapy and immunotherapy on immune mechanism.^13^ In the two cases we encountered, both patients were treated with ICI and radiotherapy, which was effective in shrinking distant metastases owing to the abscopal effect. Based on these results, the combination of ICI therapy and radiotherapy may be essential for achieving an abscopal effect.

### 3.2 Strategy for optimal abscopal effect

How should ICI be used in combination with ICI for optimal abscopal effects? Additionally, what is the best radiation procedure? Studies by Rowell et al.,^14^ Abuodeh et al.,^10^ and Hatten et al.^15^ were summarized. According to these reports, palliative radiation therapy was used in most cases, with a median total delivered dose of 31–32 Gy, median dose per fraction of 3 Gy, median time to confirmed response of 2–4 months, and median progression free survival of 6 months. In the present report, Case 1 received 20 Gy of palliative radiotherapy (4 Gy × 5 fractions), and Case 2 received 66 Gy of radical radiotherapy (2 Gy × 33 fractions), and the effect was immediately confirmed.

The optimal radiation therapy dose and fractions required for sensitization to immunotherapy have not yet been established. Preclinical data collectively suggest that 14–24 Gy in 2–3 fractions with concurrent ICI may be the optimal radiation dose, fractionation, and treatment sequence to generate a robust antitumor cytotoxic T lymphocytes response.^16^ Although hypofractionated regimens (e.g., 6 Gy × 5 fractions or 8 Gy × 3 fractions) have also been suggested to be more effective than conventional fractionation, the two cases in this study revealed that the abscopal effect is not necessarily observed only with palliative radiotherapy, but also with radical radiotherapy. However, the underlying mechanism remains unclear at present.^17^

Buchwald et al. reported that a review of the timing and dose of immunotherapy and radiotherapy indicated that a dose of nearly 10 Gy per dose in 1–3 fractions was optimal for inducing an abscopal effect.^18^ Furthermore, immunotherapy should be initiated at the start of radiotherapy; the earlier the initiation of immunotherapy, the more effective it is. In contrast, Craig et al. reported that T cells cause a systemic response at sites that do not receive radiotherapy. These effects are further enhanced by the use of immunotherapeutic agents that increase T cell activity. ICIs should be administered after radiotherapy for maximal abscopal effect.^19^ Furthermore, preclinical data from Wei et al.^20^ reported that an abscopal response was observed when αPD‐1 was administered after local irradiation of the tumor; however, abscopal activity disappeared when αPD‐1 was administered before irradiation.^20^ The results of these studies indicate that radiotherapy should be performed prior to ICI administration.

In our study, ICI were administered before radiotherapy in both cases. Currently, there is no definitive conclusion regarding the optimal sequence of treatment. Further research and the accumulation of more cases are essential to refine this combination therapy.

### 3.3 Abscopal effect in head and neck cancer

Hatten et al. have reported that the abscopal effect is common in specific cancer types.^15^ Indeed, 67% of all reports involved patients with NSCLC, renal cancer, melanoma, lymphoma, and hepatocellular carcinoma.^15^ Grimaldi et al. reported that an abscopal effect was observed in 52% (11 of 21) patients with advanced melanoma and metastases who received radiotherapy after anti‐CTLA‐4 antibody treatment.^21^ Furthermore, Gomes et al. reported that when patients with tumor growth after anti‐PD‐1 antibody therapy were treated with radiotherapy, an abscopal effect was observed in 18.7% of patients.^22^ Thus, while many cases of cancer types traditionally considered highly immunogenic have been reported, there are very few reports of abscopal effects in head and neck cancers.

Only six cases of head and neck cancer have been reported (parotid,^23^ nasal cavity,^24^, ^25^ hypopharynx,^26^ skin,^27^ and oro‐hypopharynx,^28^ respectively). Head and neck cancers are less frequent than the aforementioned cancers and are less likely to show an abscopal effect. Head and neck SCC cells have been shown to produce immunosuppressive mediators that allow them to evade the host immune system and promote metastasis.^29^ In addition, head and neck SCC is considered a “loss‐of‐function” cancer, and inactivating mutations in TP53 and CDKN2A are known to dominate the genetic background.^30^, ^31^ This is a distinct feature from “gain‐of‐function” cancers caused by activating RAS mutations, such as melanoma,^32^ and may be associated with a low abscopal effect.^31^

Both cases in this report were SCCs, with the maxillary sinus as the primary site. Two of the six cases reported in the past were nasal cavity cancers, suggesting that nasal sinus cancer is more likely to induce an abscopal effect than other types of head and neck cancers. The detailed mechanism is unknown; however, we expect that this will be important for future research and clinical practice.

### 3.4 Abscopal effect in clinical trials

Several clinical trials have investigated this topic. In head and neck cancer, a recent randomized phase II trial of nivolumab plus SBRT versus nivolumab alone in metastatic SCC of the head and neck (SRBT 9 Gy × 3 to a single lesions prior to the second ICI cycle) by McBride et al. reported that the combination of SBRT and ICIs did not improve objective response rates in non‐irradiated lesions or overall survival in unselected patients with metastatic disease.^33^ However, it is possible that this study was underpowered because of its relatively small sample size. Currently, there are limited clinical trials focused on head and neck cancer; however, clinical trials for other cancer types may contribute insights into the abscopal effect in real‐world clinical practice.

Theelen et al.^34^ reported in a pooled analysis of the PEMBRO‐RT19 (irradiation followed by ICI)^35^ and MDACC20 (simultaneous ICI)^36^ randomized trials for metastatic non‐small cell lung cancer (NSCLC) that adding radiation therapy to immunotherapy with pembrolizumab significantly increased response rates in unirradiated lesions and significantly increased progression‐free and overall survivals. Furthermore, in a phase I trial of concurrent or sequential therapy with ipilimumab, nivolumab, and stereotactic radiotherapy in patients with stage IV NSCLC, concurrent therapy was reported to be less toxic than sequential combination therapy, and multisite stereotactic body radiation therapy (SBRT) was well tolerated in patients with widely metastatic disease.^37^ Based on these findings, they stated that a combination of pembrolizumab and radiotherapy may be a treatment option for patients with metastatic NSCLC. These results need to be validated in a randomized phase III trial. In contrast, Spaas et al. conducted a randomized phase 2 trial comparing radiotherapy with ICIs and ICI monotherapy (SRBT 8 Gy × 3 to a maximum of three lesions prior to the second or third ICI cycle) for solid tumors, including head and neck cancer. The results showed no significant differences in PFS and OS.^38^ There were also no significant differences in the subgroup analyses. Because of the multi‐tumor basket trial design, the sample size per stratum was small (7 NSCLC patients, 20 SCC patients, etc.), with insufficient power to detect a difference in treatment effect between subgroups.

Additional research is required to determine the optimal dose and timing of radiotherapy, immunotherapeutic agents, and patient cohorts to adequately evaluate the possibility of abscopal effects.

### 3.5 Limitations

This case report had several limitations. The small number of cases limited the generalizability of our findings. The mechanism underlying the abscopal effect is not fully understood, and our report is based on clinical observations rather than mechanistic evidence. In addition, no biopsy of metastases was performed in either of the two cases, and the diagnosis of metastases was based on imaging findings. Furthermore, the long‐term efficacy and safety of a combination of immune checkpoint inhibitors and radiation therapy in this setting remains unclear. However, this is one of the rare cases in which an abscopal effect was observed, and this case may contribute in no small way to future elucidation of the abscopal effect.

## 4 CONCLUSION

In two cases of maxillary sinus cancer, the combination of an ICI and radiotherapy caused an abscopal effect. Although the exact mechanisms underlying the abscopal effect remain to be fully elucidated, our cases add to the clinical evidence supporting its existence and emphasize the potential of radiotherapy and immunotherapy as a combined force against metastatic head and neck cancer.

## AUTHOR CONTRIBUTIONS

**Akihiro Sakai:** Conceptualization (lead); data curation (lead); project administration (lead); resources (lead); writing – original draft (lead); writing – review and editing (lead). **Koji Ebisumoto:** Conceptualization (equal); resources (equal); data curation (equal). **Hiroaki Iijima:** resources (equal); data curation (equal). **Mayu Yamauchi:** resources (equal); data curation (equal). **Daisuke Maki:** resources (equal); data curation (equal). **Tsuyoshi Fukuzawa:** Conceptualization (equal); resources (equal); data curation (equal). **Kenji Okami:** Conceptualization (equal); project administration (equal); supervision (equal).

## CONFLICT OF INTEREST STATEMENT

The authors have stated explicitly that there are no conflicts of interest in connection with this article.

## ETHICS STATEMENT

The Institutional Review Board approved this case report (23J002), which was conducted according to the principles of the Declaration of Helsinki. We confirmed a patient's anonymity.

## PATIENT CONSENT STATEMENT

We have obtained written informed consent from the participant presented in this report.

## Data Availability

The data that support the findings of this study are available from the corresponding author upon reasonable request.
